# Drug utilization in neonatal setting of Pakistan: focus on unlicensed and off label drug prescribing

**DOI:** 10.1186/s12887-018-1211-y

**Published:** 2018-07-25

**Authors:** Muhammad Aamir, Jamshaid Ali Khan, Faisal Shakeel, Rabeea Shareef, Nazia Shah

**Affiliations:** 1 Department of Pharmacy, University of Peshawar, Peshawar, Pakistan; 2 Department of Pharmacy, Sarhad University of Science and Information Technology, Peshawar, Pakistan

**Keywords:** Neonatology, Unlicensed drug use, Off label drug use, Pakistan

## Abstract

**Background:**

Unlicensed and off label drug use is an issue recognized worldwide in pediatric pharmacotherapy. The study was designed to assess the prevalence and predictors of unlicensed and off label drug use in neonatal population of Pakistan.

**Method:**

A prospective, observation study was conducted in nursery units at pediatric department of four tertiary care hospitals during the 1 year. Micromedex DRUGDEX was used to evaluate the case notes of 1300 patients. Logistic regression was employed to calculate the odds ratio for the predictors of unlicensed and off label drug use.

**Results:**

A total of 1300 patients were included in this study who were treated with 52 different drugs. The prevalence of off label drug use was higher (52.14%) as compared to unlicensed drug use (33.35%). Dose (61.29%) and indication (13.68%) were the most frequent reasons for off label prescribing. In comparison to the corresponding reference categories, females and preterm infants were less likely to receive unlicensed prescriptions. While patients staying at hospital more than 5 days and infants receiving 3 or more medications were significantly more likely to receive unlicensed prescriptions. Moreover, in comparison to the corresponding reference categories, females were less likely to receive off label prescriptions while infants receiving 3 or more medications were 7 times more likely to receive off label prescriptions.

**Conclusion:**

The significant prevalence of unlicensed and off label drug prescriptions was found in neonatal population of Pakistan. The findings imply that more data on prevalence of unlicensed and off label prescriptions are required to provide a better picture of pediatric therapy in developing countries. Furthermore, advance formulations with new dosing in pediatrics is also necessary to minimize the risk of adverse drug events.

**Electronic supplementary material:**

The online version of this article (10.1186/s12887-018-1211-y) contains supplementary material, which is available to authorized users.

## Background

Challenges faced by healthcare professionals are unique in pediatric population as compared to that of adults, as varied drug response can occur in the absorption, distribution, metabolism, and elimination, affecting the efficacy or safety of the drug. [1]. To combat these challenges, certain steps have been taken in the past two decades by Food and Drug Dosage and European Union for pediatric pharmacotherapy but still neonates face issues of unlicensed and off label prescription [2].

Unlicensed drug refers to employment of a drug in lack of product license, while off label drug refers to drug prescribed outside the specifications labelled on the product license [3]. Lack of controlled clinical trials in neonates, low funding by local governments and lack of interest of drug manufacturers (due to less profit and difficulty in development of pediatric formulations) are the pivotal reasons for frequent unlicensed and off label drug prescribing in neonates [4]. Unlicensed and off label drug use may lead to serious adverse drug reactions (ADRs) as reported by several studies [5, 6]. The risk of ADRs is increased two folds in neonates because of their physiological immaturity [7].

To date, no national or regional surveys have been conducted in Pakistan to evaluate unlicensed and off label drug use in neonates. The study was designed to assess the prevalence and predictors of unlicensed and off label prescriptions.

## Methods

Data for this study was collected from each nursery at pediatric department of four tertiary care hospitals in Peshawar, Pakistan. Nursery was the second most populous ward after medical unit in our region. The nursery of private hospital (NWGH) was better facilitated as compared to government hospitals (HMC, KTH and LRH). However, number of beds were more in government hospitals than in private hospital. No neonatal intensive care unit was present in any of the hospitals and all neonates who required critical care were treated in the nurseries of the respective hospitals.

The prospective observational study was conducted from 15th May, 2014 to 15 April, 2015. Sample size was determined using an appropriate formula for known populations. A total of 1345 case notes were collected, in order to complete sample size (1300 patients were included which fulfil our inclusion criteria). Cluster sampling technique was employed to collect the data. Primary data was collected on predesigned proforma. Data source was the patient medication charts which provided information of identification number, name, age, gender, duration of stay, diagnosis and prescribed medications. Patients admitted to nursey unit with a minimum duration of stay of 24 h in the hospital were included in the study. Topical medications, oxygen therapy and IV’s stock solutions were not considered for evaluation.

Age groups of neonates were categorized on the basis of recommended World Health Organization (WHO) guidelines. Patients of age less than 38 weeks were considered as preterm, while those of age above 38 weeks were categorized as infants [8]. Morbidities were classified according to International classification of diseases (ICD) 10 system [9]. Anatomical Therapeutic Chemical (ATC) classification system was used to classify drugs [10]. Patient case notes were evaluated using Thomson Healthcare Micromedex DrugDex database [11]. Our variables of interest were unlicensed and off label prescriptions. Prevalence and predictors for both the variables were analyzed. In current study unlicensed drugs were consider those drugs which were unlicensed for neonates by FDA as mentioned in reference database. Off label prescriptions were assessed on basis of following categories (1) Age (2) Indication (3) Dose (4) Dosage form (5) Age-Indication (6) Age-Dose (7) Age-Dosage form (80 Indication-Dose (9) Dose-Dosage form (10) Indication-Dosage form (11) Age-Indication-Dose (13) Age-Indication-Dosage form (14) Age-Dose-Dosage form (15) Indication-Dose-Dosage form.

Age, gender, number of prescribed drugs, duration of stay, unlicensed and off label drugs use were summarized using frequencies. Unlicensed and off label prescriptions were dependent variables and presented as (*Y* = 1, *N* = 0) in model. Multivariate binary logistic regression was employed to determine the odds ratio with 95% confidence interval (CI) for the predictors of dependent variables. Age, gender, duration of stay and number of prescribed drugs were independent variables in model and considered as predictors [12]. Analysis was performed using IBM SPSS Statistics for Windows, version 20. (Armonk, NY: IBM Corp.). Chi square was used to determine the association of unlicensed and off label drug use with age groups and hospitals.

## Results

### Demographics

A total of 1300 patients were included from the nursery of four hospitals, of which 350 (26.92%) were from HMC, 240 (18.46%) were from NWGH, while 355 (27.31%) were from KTH and LRH each. Out of total neonates, 271 (20.76%) were born with premature status in nursery unit of four tertiary care hospitals. Median gestational age for preterm babies was 33 weeks (range 26 to 35 week). The male to female ratio was 17:8. The mean age of the population was 8 (±18.45) days and age of the patients ranged from 1 day to 1 month. Of the total patients, 74.92% patients were between the ages of 1 to 7 days, 11.00% were between the age of 8 to 14 days, 3.54% were between the ages of 15 to 21 days and 10.54% were 22 to 30 days. Mean number of prescribed drugs were 2.85 (±1.358). While Mean duration of stay in the nurseries was 3.15 (±2.8) days.as shown in Table 1.

**Table 1:** General characteristics of patients in nurseries

Variables	n (%) HMC	n (%) KTH	n (%) LRH	n (%) NWGH	Total
Gender					
Male	253 (19.46)	232 (17.85)	236 (18.15)	163 (12.54)	884 (68.00)
Female	97 (7.46)	123 (9.46)	119 (9.15)	77 (5.92)	416 (32.00)
Total	350 (26.92)	355 (27.31)	355 (27.31)	240 (18.46)	1300 (100)
Gestational age					
Term Infants	272 (20.92)	256 (19.69)	294 (22.62)	207 (15.92)	1029 (79.15)
Preterm infants	78 (6.00)	99 (7.62)	61 (4.69)	33 (2.54)	271 (20.85)
Total	350 (26.92)	355 (27.31)	355 (27.31)	240 (18.46)	1300 (100.00)
Age (Days)					
≤ 7 days old	275 (21.15)	280 (21.54)	309 (23.77)	110 (8.46)	974 (74.92)
8 to 14 days old	25 (1.92)	35 (2.69)	41 (3.15)	42 (3.23)	143 (11.00)
15 to 21 days old	20 (1.54)	12 (0.92)	5 (0.38)	9 (0.69)	46 (3.54)
22 to 30 days	30 (2.31)	28 (2.15)	–	79 (6.08)	137 (10.54)
Total	350 (26.92)	355 (27.31)	355 (27.31)	240 (18.46)	1300 (100)
Mean ± S.D	0.20 **±** 0.327	0.21 ± 0.36	0.10 **±** 0.111	0.69 ± 1.20	0.27 ± 0.613
Range	0.97 (0.03–1.00)	2.97 (0.03–3.00)	0.47 (0.03–0.05)	0.97 (0.03–1.00)	0.97 (0.03–1.00)
Prescribed drugs					
1 to 3 drugs	255 (19.62)	326 (25.08)	257 (19.77)	148 (11.38)	986 (75.85)
4 to 6 drugs	93 (7.15)	21 (1.62)	94 (7.23)	78 (6)	286 (22)
7 and above drugs	2 (0.15)	8 (0.62)	4 (0.31)	14 (1.08)	28 (2.15)
Total	350 (26.92)	355 (27.31)	355 (27.31)	240 (18.46)	1300 (100)
Mean ± S.D	2.99 ± 1.193	2.43 ± 1.096	2.94 ± 1.3	3.10 ± 1.765	2.85 ± 1.358
Range	6 (2–8)	7 (1–8)	7 (1–8)	8 (1–9)	8 (1–9)
Hospital stay					
1 to 5 days	331 (25.46)	317 (24.38)	311 (23.92)	175 (13.46)	1134 (87.23)
6 to 10 days	19 (1.46)	32 (2.46)	44 (3.38)	46 (3.54)	141 (10.85)
11 or more days	–	6 (0.46)	–	19 (1.46)	25 (1.92)
Total	350 (26.92)	355 (27.31)	355 (27.31)	240 (18.46)	1300 (100)
Mean ± S.D	2.14 ± 1.395	2.43 ± 1.096	3.50 ± 1.606	4.60 ± 4.882	3.15 ± 2.800
Range	7 (1–8)	16 (1–17)	9 (1–10)	34 (1–35)	34 (1–35)
Patients received at least one unlicensed drug					
Y	254 (19.54)	290 (22.31)	252 (19.38)	205 (15.77)	1001 (77)
N	96 (7.38)	65 (5.00)	103 (7.92)	35 (2.69)	299 (23)
Total	350 (26.92)	355 (27.31)	355 (27.31)	240 (18.46)	1300 (100)
Patients received at least one off label drug					
Y	320 (24.62)	294 (22.62)	252 (19.38)	150 (11.54)	1016 (78.15)
N	30 (2.31)	61 (4.69)	103 (7.92)	90 (6.92)	284 (21.85)
Total	350 (26.92)	355 (27.31)	355 (27.31)	240 (18.46)	1300 (100)

The primary diagnosis of the neonates was neonatal sepsis (18.23%), followed by hypoxic ischemic encephalopathy (17.92%), neonatal jaundice (15.00%), preterm observation (9.08%), respiratory distress syndrome (7.15%), birth asphyxia (5.46%), meconium aspiration syndrome (3.23%), pneumonia (2.92%), meningitis (2.77%) and kernicterus (2.31%).

#### Prescribed medicines and their therapeutic categories

Anti-infective for systemic use (72.97%) and central nervous system (12.3%) were prevalent therapeutic drug categories in nursery units of pediatric department of four tertiary care hospitals. Commonly prescribed medications in all the four hospitals were ampicillin (29.23%), ceftriaxone (28.57%), amikacin (5.83%), phenobarbitone (5.28%), ceftazidime (5.16%), phenytoin (2.32%), gentamicin (2.26%), captopril (2.03%), Paracetamol (1.94%), and aminophylline (1.80%).

#### FDA unlicensed and off label drug use

**Fig. 1:**
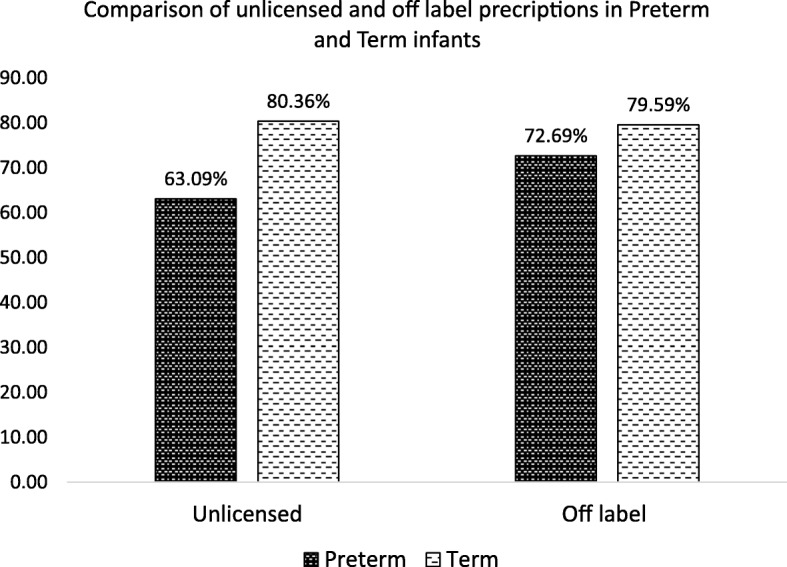
Comparison of unlicensed and off label prescriptions in preterm and term infants

A total of 52 drugs were prescribed 3448 times, of which 1150 (33.35%) were unlicensed and 1798 (52.14%) were off label. Out of the total patients, 1001 (77.00%) received at least one unlicensed drug and 1016 (78.15%) received at least one off label drug. The detail of patients received at least one unlicensed and one off label drug is given in Table 1. Comparison of preterm and term infants regarding unlicensed and off label drug use are displayed in Fig. 1.

#### Off label categories

**Fig. 2:**
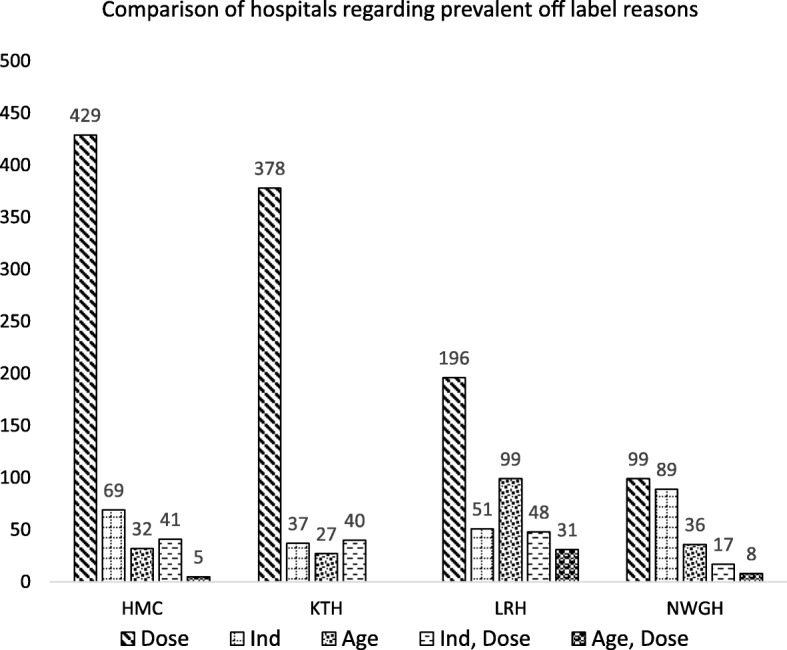
Comparison of hospitals regarding prevalent off label reasons

Dose (61.29%) and indication (13.68%) were the most frequent off label categories in all the therapeutics classes. Other observed off label categories were; age (10.79%), indication-dose (8.12%), age-dose (2.45%), indication-dosage form (1.67%), age-dosage form (0.67%), dosage form (0.50%), dosage form-dose (0.50%), age-indication (0.11%), age-indication-dose (0.11%) and age-dosage from-dose (0.11%). %). The commonly prescribed off label drugs along with their categories are shown in Table 2. Distribution of prevalent off label drug categories among hospitals are presented in Fig. 2.

**Table 2:** Most prevalent prescribed off label medicines with their off label categories

Drugs	Age	Indication	Dose	Dosage form	Age- Dose	Indication- Dose	Indication- Dosage form	Dosage form- Dose	Total
Ampicillin	–	156	906	–	–	109	–	–	1171
Cefotaxime	–	152	854	–	–	105	–	–	1111
Phenobarbitone	–	95	126	2	–	99	34	4	360
Ceftazidime	–	–	222	–	–	–	–	–	222
Amikacin	–	–	194	–	–	–	–	–	194
Phenytoin	–	19	106	5	–	17	–	–	147
Captopril	60	–	58	–	11	–	–	–	129
Paracetamol	78	–	52	–	11	–	–	–	141
Gentamicin	–	16	55	–	–	12	–	–	83
Aminophylline	–	64	60	–	–	13	–	–	137

#### Association of unlicensed drug use with hospitals and age groups

Chi square value determine that unlicensed drug use was significantly associated with the type of hospital (*p* < 0.0001). Further indicating that prevalence of unlicensed drug prescribing was high in the private setup (NWGH-85.41%, 205/240 patients) as compared to government setups (HMC-72.57%, 254/350 patients; KTH-81.69%, 290/355 patients and LRH-70.98%, 252/355). A significant association was also found between unlicensed drugs prescribing with age groups (*p* < 0.0001). Unlicensed drugs were prescribed in 72.94% of 7 days old, in 88.81% of 8 to 14 days old, 95.96% 15 to 21 days old and in 86.86%% in 22 days and above category.

#### Association of off label drugs use with hospitals and age groups

There was significant association between off label drug use and type of hospitals (*p* < 0.001) when analyzed using chi square. Off label drug prescribing was lesser in private hospital as compared to government hospitals i.e.*,* government hospitals; in HMC-91.42%, in KTH-82.81%, in LRH-70.98%, and private hospital; in NWGH-62.5%. Medications prescribed off label was insignificantly associated with age groups (*p* < 0.332). Drugs prescribed off-label were more frequently found in children (49.7%, 445/895 patients) and infants (23.4%, 210/895 patients).

### Predictors for unlicensed and off label prescriptions

Multivariate binary logistic regression showed that females (OR 0.743, CL 0.560–0.985) were significantly less likely to receive unlicensed prescriptions as compare to males. Similarly, preterm infants (OR 0.537, CL 0.396–0.729) were significantly less likely to receive unlicensed drugs as compared to term infants. While patient staying at hospital more than 5 days (OR 1.730, CL 1.037–2.888) were 1.7 times significantly more likely to receive unlicensed medications as compared to patients staying at hospital less than 5 days. Infants receiving 3 or more medications (OR 3.360, CL 2.548–4.592) were also 3.3 times significantly more likely to receive unlicensed prescriptions as compared to reference category.

While evaluating off label prescriptions, females (OR 0.720, CL 0.539–0.962) were significantly less likely to receive off label prescriptions as compare to males. Infants receiving 3 or more medications (OR 7.187, CL 4.961–10.411) were 7 times more likely to receive off label drugs as compare to patients receiving 1 or 2 drugs as shown in Table 3.

**Table 3:** Predictors of unlicensed and off label prescriptions in nursery wards

S.No.	Variables			*p-* value	OR (95% CI)
		Unlicensed prescriptions			
		Present (*n* = 1001)	Absence (*n* = 299)		
1	Gender				
	Female	302	114	0.039^**^	0.743 (0.560–0.985)
	Male	699	185		Reference
2	Gestational age				
	Preterm infants	174	97	0.000^***^	0.537 (0.396–.729)
	Term infants	827	202		Reference
3	Length of Stay				
	5 or More days	148	20	0.036^**^	1.730 (1.037–2.888)
	Less than 5 days	855	279		Reference
4	Prescribed drugs				
	3 or More drugs	521	64	0.000^***^	3.360 (2.548–4.592)
	Less than 2 drugs	480	235		Reference
		Off label prescriptions			
		Present (*n* = 1016)	Absence (*n* = 284)		
1	Gender				
	Female	304	112	0.026^**^	0.720 (0.539–0.962)
	Male	712	172		Reference
2	Gestational age				
	Preterm infants	179	76	0.854	0.970 (0.700–1.343)
	Term infants	837	208		Reference
3	Length of Stay				
	5 or More days	148	20	0.083^*^	0.662 (0.415–1.055)
	Less than 5 days	855	279		Reference
4	Prescribed drugs				
	3 or More drugs	544	41	0.000^***^	7.187 (4.961–10.411)
	Less than 2 drugs	472	243		Reference

*OR* Odds Ratio, *CI* Confidence interval

^*^significant at *p < 0.1* ^**^significant at *p < 0.05* ^***^significant at *p < 0.01*

## Discussion

In Neonates, proportion of unlicensed prescriptions (33.35%) were comparatively less as compared to off label prescriptions (52.14%). A Turkish study conducted in neonatal intensive care revealed a high prevalence (62.3%) of unlicensed or off label drug use in neonates [13]. In contrast a study conducted over a period of 13 weeks in England reported 10% of unlicensed prescriptions [14]. Similar results were obtained by a French study when they analyzed 40 new born for unlicensed prescriptions (10%) [15]. A Spanish report also showed low prevalence of unlicensed prescriptions (13%) in neonates [16]. An Irish report revealed low prevalence (19%) of unlicensed drug use from neonatal intensive care unit [17]. These results demonstrate that there are legal differences in the market authorization of medicines and clinical practice among countries. In addition, results clearly shows the difference of developed and developing countries. More awareness and research studies are required in developing countries about unlicensed drug use among prescribers and health care professionals to enhance the quality of pediatric regimen.

An Irish and Australian studies reported 39 and 47% of off label prescriptions which is low as compared to our results [17, 18]. The prevalence of off label prescriptions was 54.7 and 50% in England and Spain which is concomitant to our findings [14, 16]. While a higher prevalence was also reported from France regarding the use of off label prescriptions 63% [15]. A Multicenter study conducted in Italy included 220 newborn infants, which also revealed a high prevalence of off label prescriptions (73.5%) [19]. The variations in results is because the current study was conducted in a much larger population compared to others studies and also due to variation of clinical practice among pediatricians in nursery unit.

Dose (61.29%) was observed to the most common reason for off label drug use. A similar reason for off label drug use was reported by many other studies [18–20]. Off-label prescriptions due to low or high doses were demonstrated by other studies; as low dose cannot achieved the desired outcome of therapy and an overdose can cause a higher risk of toxicity [14, 19, 21]. For example, faster metabolism of cyclosporin in pediatrics could lead to subtherapeutic plasma level because of under-dosing [22].

Anti-infective for systemic use (51.37%) and drugs used in respiratory system were the frequently prescribed therapeutic drug categories. A multicenter study performed at NICU in Italy reported similar findings [19]. Another multicenter report published in Italy, based on 4265 prescriptions also observed anti-infective for systemic use as the most prevalently prescribed therapeutic drug category [20]. A Turkish research conducted in 17 NICU also reported concurrent findings [13]. A prospective cohort study performed in Australia also comply with our results [18]. The frequent use of anti-infective for systemic use and drugs used in respiratory system are due to high neonatal risk to microbial infections and their immature respiratory system especially in preterm babies.

Two antibiotics ampicillin and cefotaxime were the most frequently prescribed in neonatology units. Warrier et al., conducted a study at Detroit, United states reported concomitant findings [23]. However, a report published by Clark et al., showed that combination of ampicillin/gentamicin was more effective as compared to ampicillin/ cefotaxime in newborns infants for infectious diseases [24]. Ampicillin was more frequently prescribed drug in neonates and was also reported by a Portuguese research [25]. Many other studies conducted in different regions also reported ampicillin as most commonly prescribed drug [13, 19, 26].

Preterm neonates, patients receiving 3 or more medications, infants staying at hospital more than 5 days and female gender were significant risk factors for unlicensed drug use while preterm infants and infants receiving 3 or more medications were significant risk factors for off label prescriptions in current study. Gender is not taken into consideration during the drug development [12]. However, predominant ratio of males in the current study might be the reason of female significance in the statistical mode. Preterm and neonates in first week of life are more exposed to unlicensed and off label prescriptions because of limited scope of regimens available in particular age groups. The primary reason for limited scope of therapies in neonates are lack of clinical drug trials [18]. Another reason may be the ethical approval of these drug trials. Pharmaceutical drug trials in pediatrics is also a controversial topic throughout the world [18, 27]. It is often a necessity to use unlicensed or off-label drugs in neonates that have not been tested clinically for their respective age, indications, doses, or route of dosage.

Pakistan follows the both the Food and Drug Dosage guidelines and European Union Regulation On Medicinal Products for Pediatric Use (Regulation n° 1901/2006) implemented in 2007. European Medicinal Agency (EMA) published a report on clinical experience obtain with the application of this module which introduced and implemented a system of obligations, incentives and rewards to the development of medicines for pediatric age in European Member States [28]. The report included 600 pediatric investigations. Plans approved by the end of 2012, most of them for drugs that were not authorized in European Union and the remaining related to new morbidities for patent-protected products or pediatric-use marketing authorizations. However, only 2% of medicines were entirely referred to neonatal intensive care [29].

The number of pediatric clinical drug trial remained constant between 2006 and 2012, but there was a sophisticated upsurge in the number of pediatric studies, in particular for the infant age group, who were normally not considered in the clinical trials before 2008. More than 18,000 reports were submitted to European medicine agency since 2008 regarding the use of medicine in pediatrics, but it originated only 65 changes in approved products [29]. These outcomes are encouraging, but it is still a long procedure until we attain satisfactory and ideal pediatric pharmacotherapy.

Neonates with stay less than 24 h were not included in this study. Further studies are also required in other pediatric units at tertiary care hospitals in our country. So the results should be generalized with caution in population.

## Conclusion

The prevalence of unlicensed and off label drug use was high in neonates and not reported in Pakistan till date. Physicians must be reminded of the unlicensed and off label drug use when prescribing medications for newborns. Suitable medical interventions must also be established by drug manufactures and government agencies for betterment of neonatal pharmacotherapy.

## Additional file

Additional file 1: Nursery all cases. File contains all the data which has been analyzed for this manuscript. (XLSX 584 kb)

## Abbreviations

GCP: Good Clinical Practice
HMC: Hayatabad Medical Complex
KTH: Khyber Teaching Hospital
LRH: Lady Reading Hospital
NWGH: Northwest General Hospital
